# Necroptosis in stressed ovary

**DOI:** 10.1186/s12929-019-0504-2

**Published:** 2019-01-21

**Authors:** Govind R. Chaudhary, Pramod K. Yadav, Anil K. Yadav, Meenakshi Tiwari, Anumegha Gupta, Alka Sharma, Ashutosh N. Pandey, Ajai K. Pandey, Shail K. Chaube

**Affiliations:** 1 Cell Physiology Laboratory, Department of Zoology, Institute of Science, Banaras Hindu University, 221005, Varanasi, India; 2 Department of Kayachikitsa, Faculty of Ayurveda, Institute of Medical Science, Banaras Hindu University, 221005, Varanasi, India

**Keywords:** Ovary, Stress, Oxidative stress, Necroptosis, RIPK, MLKL, TNF

## Abstract

Stress is deeply rooted in the modern society due to limited resources and large competition to achieve the desired goal. Women are more frequently exposed to several stressors during their reproductive age that trigger generation of reactive oxygen species (ROS). Accumulation of ROS in the body causes oxidative stress (OS) and adversely affects ovarian functions. The increased OS triggers various cell death pathways in the ovary. Beside apoptosis and autophagy, OS trigger necroptosis in granulosa cell as well as in follicular oocyte. The OS could activate receptor interacting protein kinase-1(RIPK1), receptor interacting protein kinase-3 (RIPK3) and mixed lineage kinase domain-like protein (MLKL) to trigger necroptosis in mammalian ovary. The granulosa cell necroptosis may deprive follicular oocyte from nutrients, growth factors and survival factors. Under these conditions, oocyte becomes more susceptible towards OS-mediated necroptosis in the follicular oocytes. Induction of necroptosis in encircling granulosa cell and oocyte may lead to follicular atresia. Indeed, follicular atresia is one of the major events responsible for the elimination of majority of germ cells from cohort of ovary. Thus, the inhibition of necroptosis could prevent precautious germ cell depletion from ovary that may cause reproductive senescence and early menopause in several mammalian species including human.

## Background

Stress has affected physical, social and psychological status of a person in the modern society [1, 2]. Although both genders are exposed to various kinds of stressors, females are more frequently exposed to one or other type of stressors during their reproductive life [3–5]. Several factors such as lifestyle, pressure and demands may generate psychological stress [2]. The psychological stress triggers the release of cortisol and generation of reactive oxygen species (ROS) in the body. Further, accumulation of ROS in the ovary results in oxidative stress (OS) [1, 6]. Studies suggest that high level of cortisol as well as OS induce granulosa cell death [2, 6, 7]. The granulosa cell death deprives follicular oocytes from nutrients, growth factor, survival factors and reduces estradiol biosynthesis [6]. The reduced level of estradiol-17β affects folliculogenesis and deteriorates oocyte quality by inducing various cell death pathways in somatic cells as well as in follicular oocyte [6–8]. Studies suggest that estradiol-17β could act as an antioxidant [9, 10] and protect OS-mediated apoptosis in pig [11] and ovine follicles [10, 12]**.** Although ovary is a dynamic organ and has its own antioxidant enzymes to scavenge ROS during final stages of folliculogenesis, depletion of antioxidants system could result in the accumulation of ROS and thereby OS in the ovary [13].

ROS affects oocyte physiology by modulating meiotic cell cycle resumption/arrest and cell death depending upon its level [6, 7, 14–21]. For instance, a moderate level of ROS triggers oocyte meiotic resumption from diplotene as well as M-II arrest [19, 22], while supplementation of antioxidants inhibits spontaneous resumption under in vitro culture condition [16, 17, 23]. Further, high level of ROS generates OS and induces meiotic cell cycle arrest and thereby apoptosis in rat oocytes cultured in vitro [6–8, 24–27]. The extremely high level of ROS induces necrosis in oocytes of several mammalian species including mouse [28], rat [29], ewe [30] and human [31].

Necrosis is morphologically characterized by organelle swelling, increase of cell volume and rupture of cell membrane [32]. Studies suggest that regulated form of necrosis so called necroptosis shows morphological features similar to necrosis [33]. A few studies indicate the occurrence of OS-mediated necroptosis in cow [34] and human ovary [35]. The OS-mediated necroptosis in granulosa cells and oocyte remains ill understood. This review article updates the information on stress-mediated necroptosis and proposes a possible molecular mechanism underlying OS-mediated necroptosis in mammalian ovary.

### Stress and necroptosis in granulosa cells

Increase of ROS in the follicular fluid under physiological range is beneficial for follicular oocyte. For instance, a moderate increase of ROS is associated with spontaneous meiotic resumption, fertilization rate and reproductive outcome in rat [16] and human [16, 17, 23]. However, sustained high level of ROS generates OS and increased OS trigger granulosa cell death in rat [6, 7, 13]. The possible source for the increased level of ROS in the follicular fluid seems to be macrophages and the extracellular ROS together with TNF-α produced by macrophages, may trigger necroptosis of encircling granulosa cells [34]. The granulosa cell death subsequently starves oocyte and results in more vulnerable to cell death. The elevated intracellular ROS would trigger apoptosis, necrosis or necroptosis in response to the extent of insult and different stress conditions. In addition, ROS is cell permeable and it can easily enter in granulosa cells from follicular fluid. Thus, it is not possible to distinguish the necroptosis triggered by extracellular or intracellular ROS within the follicular microenvironment.

The prolonged starvation causes generation of ROS and induces necroptosis in human granulosa cells [35]. The increased level of ROS has been reported to inhibit cleavage of caspase and result in necroptosis in human ovary [33, 35]. The high level of ROS increases receptor interacting protein kinase 1 (RIPK1) and receptor interacting protein kinase 3 (RIPK3) in human granulosa cells [35]. Dehydroepiandrosterone (DHEA) reduces ROS production and RIPK expression in starvation-mediated necroptosis in human granulosa cells [35]. DHEA has been reported to improve oocyte quality probably by its antioxidant property [36]. The OS increases the expression of few enzymes that induce necroptosis in human granulosa cells [37]. Read-through acetylcholinesterase (AChE-R) is a stress form of acetylcholinesterase (AChE) and its expression increases in response to OS [38, 39]. The acetylcholine (ACh) is cleaved by AChE-R and triggers granulosa cell necroptosis in human [37]. The phosphorylated mixed lineage kinase domain-like protein (p-MLKL) has been reported during necroptosis in mouse and human ovary [40]. This is supported by the observation that the p-MLKL triggers granulosa cell necroptosis. The use of necrosulfonamide reduces necroptosis in human granulosa cells cultured in vitro [37, 41].

The increased number of macrophages has been reported during luteolysis in bovine corpus luteum [34, 42] and produce tumor necrosis factor alpha (TNF-α) as well as interferon-γ (IFNG) [34, 43]. The high level of cytokines triggers expression of RIPK1 and RIPK3 in luteal cell of cow ovary [34], human T-cells [44] and mouse dendritic cells [45]. The RIPK1 and RIPK3 act as stress sensors that promote necroptosis in cow ovary [34, 46]. On the other hand, necrostatin-1 treatment inhibits TNF-α and IFNG-mediated increase of RIPK-1 as well as RIPK-3 expression in granulosa cells of cow ovary [34]. These studies suggest that the increase of ROS generate OS in the follicular fluid of ovary. The OS triggers necroptosis in encircling granulosa cells through RIPK as well as MLKL-signaling pathways. The OS-mediated granulosa cell necroptosis could increase susceptibility of follicular oocyte in several mammalian species including human.

Environmental pollutants including cadmium (Cd) have been reported to trigger necroptosis in chinese hamster ovary (CHO) cells [47]. The Cd exposure increases intracellular calcium [(Ca^2+^)i] and generation of ROS and thereby calpain activity [47, 48]. Increased calpain activity reduces mitochondrial membrane potential (MMP), while increased level of ROS inhibits NF-κB activity [47]. The reduced level of MMP and NF-κB activities lead to Cd-induced necrotic cell death possibly through necroptosis. This possibility is further supported by the observations that the specific inhibitor of necroptosis, necrostatin-1, attenuates Cd-induced necroptosis by restoring the NF-κB activity in CHO cells [47].

### Stress and necroptosis in oocyte

The endogenous burst of internal calcium stores result in extremely high level of [(Ca^2+^)i] that induce necrotic cell death through the generation of ROS in oocyte [22]. The follicular oocyte secretes GDF-9 that helps in the cumulus cell expansion and granulosa cell proliferation [49–51]. The possible involvement of necroptosis in oocyte comes from the observations that the necrostatin-1 treatment increases GDF-9 and mitotic arrest deficient 2 (Mad2) expressions required for meiotic competency of mouse oocyte [49]. Further, necrostatin-1 also increases Bcl-2 expression that ensures oocyte survival in mouse [49]. In addition, necrostatin-1 prevents OS-mediated deterioration of oocyte quality in mouse by modulating RIPK1 activity [49].

Immune suppression causes necrotic cell death in follicular oocyte and depletes ovarian reserve in mouse [52]. The death of oocyte under compromised immune system could be a programmed necrosis due to the involvement of a cytokine such as TNF-α [53, 54]. The TNF-α binds to it’s receptor and triggers necroptosis through RIPK1-mediated pathway [33, 55]. The increased TNF-α attenuates the development of ovary [56], decreases number of mature follicles in ovary and impairs oocyte meiotic maturation in mouse [52]. This is further supported by the observation that inhibition of TNF-α protein synthesis prevents spontaneous meiotic maturation in mouse oocyte cultured in vitro [52]. Thus, ROS-mediated TNF-α signaling may induce necroptosis in follicular oocytes in the ovary as described in granulosa cells.

**Fig. 1:**
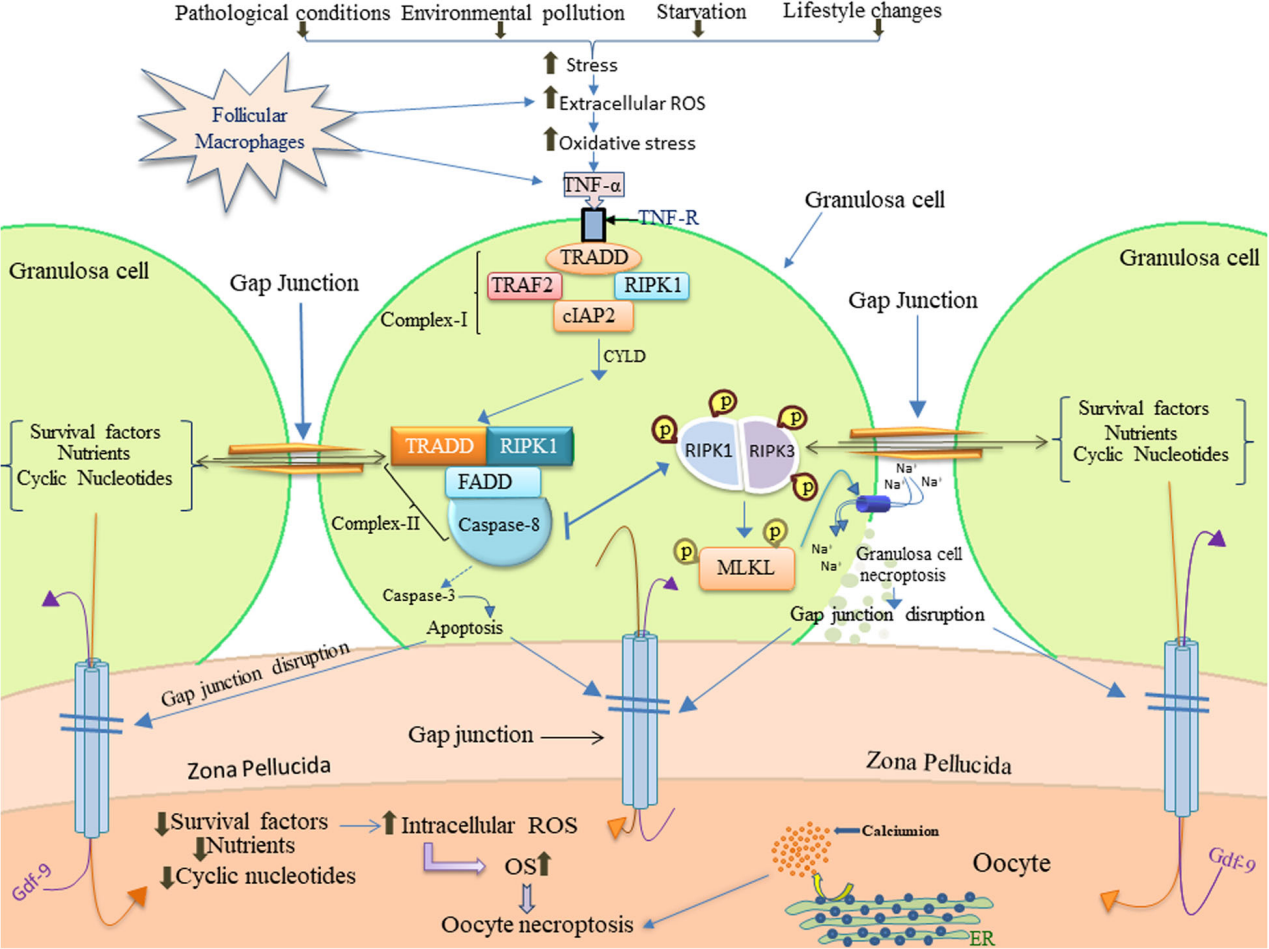
Pathological conditions, environmental pollutants, starvation and lifestyle changes generate stress in the body. Stress increases extracellular ROS production from macrophages, OS and cytokines level in the granulosa cell of mammalian ovary. Stress as well as enhanced OS generates TNF-α that binds to its receptor present on the granulosa cell membrane and induces conformational changes that results a binding of TRADD with death domain of a receptor. TRADD recruits RIPK1 as well as TRAF2 forming complex-I. CYLD deubiquitinates RIPK1 allowing complex-I to dissociate from membrane. Complex-I moves into cytoplasm and associates with FADD as well as caspase-8 forming complex-II. Complex-II is responsible for the induction of apoptosis. Inhibition of caspase-8 allows the formation of necrosome and association of RIPK1 with RIPK3 that induce autophosphorylation of RIPK1 as well as RIPK3. RIPK1-RIPK3 complex phosphorylates MLKL that triggers damage of cell membrane resulting in necroptosis. Granulosa cell death deprives oocyte from survival factor, nutrients and cyclic nucleotides that lead to generation of ROS and thereby OS. The OS as well as increased level of intracellular calcium triggers oocyte necroptosis following a similar pathway as described for granulosa cell necroptosis

Oocyte is one of the major sources for TNF-α [57, 58] and its receptors are reported on oocyte as well as granulosa cells of rat ovary [58]. TNF-α binds to its receptor (TNFR) and triggers trimerization of cytoplasmic domain of TNFR1 containing TNFR1-associated death domain protein (TRADD). The TRADD binds to RIPK1 as well as TNFR associated factor 2 (TRAF2) forming complex-I [59, 60]. Cylindromatosis (CYLD) deubiquitinates RIPK1 [60, 61] and helps in the dissociation of complex-I from plasma membrane. The complex-I get associated with Fas-associating protein with death domain (FADD) and caspase-8 forming complex-II in the cytoplasm [62, 63]. Caspase-8 cleaves RIPK1 and RIPK3 resulting in apoptotic signals [55, 60, 64–66]. On the other hand, inhibition of caspase-8 results in RIPK1 and RIPK3 association and their autophosphorylation induces necroptosis [67, 68]. Activated RIPK3 then phosphorylates MLKL that finally leads to rupture of plasma membrane [40, 60, 69–72] (Fig. 1). The plasma membrane rupture is one of the important morphological features of necroptosis. The disruption of transfer of nutrients and signal molecules from dying granulosa cells to the oocyte is sufficient to trigger necroptosis. Further, nutrient deprivation may generate ROS in the oocyte and calcium burst from internal stores may trigger TNF-α signaling to induce oocyte necroptosis. The cross-talk between granulosa cells and oocyte decide the fate of each other, hence it is not yet clear which pathway is responsible for oocyte necroptosis.

### Beneficial impact of necroptosis in pathological ovary

Growing body of evidences suggest the beneficial role of necroptosis in controlling tumor growth in pathological ovary [73]. The majority of patients (60–85%) respond to primary therapy initially but later disease recurrence has been observed [74, 75]. This could be due to the evasion of apoptosis [76]. Studies suggest that the inhibitors of apoptosis protein (IAP) antagonism triggers necroptosis in apoptosis-resistant ovarian cancer cell [73]. It has been observed that necroptosis selectively occurs in apoptosis resistant cells [73]. The oncolytic vaccinia virus has been used to induce programmed necrosis in ovarian cancer cell [77]. The inhibition of RIPK1 and its substrate MLKL attenuate ovarian cancer cell death [77]. This is supported by the observations that the inhibition of RIPK1 and MLKL protect from vaccinia-mediated necroptosis in ovarian cancer cell [78]. These studies suggest that the induction of necroptosis in pathological ovary having ovarian cancer could be beneficial to control the cancer cell proliferation and may be used in the therapeutic design of ovarian cancer management [73].

## Conclusions

Stress is frequently observed at each level of society and affecting day to day as well as social life of a person. Stress not only increases the cortisol production but also induce generation of ROS. High level of ROS causes OS that negatively affects physiology of mammalian ovary. An increased OS induces granulosa cell and oocyte necroptosis by operating RIPK as well as MLKL-mediated signaling pathways. Although necroptosis could be beneficial to prevent tumor growth in pathological ovary, its involvement in granulosa cell and oocyte may cause precautious depletion of germ cells from the cohort of ovary. Thus, prevention of necroptosis in normal and healthy ovary may prevent reproductive senescence as well as early menopause in several mammalian species including human.

## Abbreviations

ACh: Acetylcholine
AChE: Acetylcholinesterase
AChE-R: Read-through Acetylcholinesterase
Cd: Cadmium
CHO: Chinese hamster ovary
DHEA: Dehydroepiandrosterone
FADD: Fas associating protein with death domain
GDF9: Growth differentiation factor 9
IAP: inhibitors of apoptosis protein
IFNG: interferon γ
Mad2: Mitotic arrest deficient 2
MLKL: Mixed lineage kinase domain like protein
MMP: Mitochondrial membrane potential
OS: Oxidative stress
RIPK1: Receptor interacting protein kinase-1
RIPK3: Receptor interacting protein kinase-3
ROS: Reactive oxygen species
TNFR: Tumor necrosis factor receptor
TNF-α: Tumor necrosis factor alpha
